# New Tools for Systematic Evaluation of Teaching Qualities of Medical Faculty: Results of an Ongoing Multi-Center Survey

**DOI:** 10.1371/journal.pone.0025983

**Published:** 2011-10-14

**Authors:** Onyebuchi A. Arah, Joost B. L. Hoekstra, Albert P. Bos, Kiki M. J. M. H. Lombarts

**Affiliations:** 1 Department of Epidemiology, UCLA School of Public Health, University of California Los Angeles, Los Angeles, California, United States of America; 2 UCLA Center for Health Policy Research, Los Angeles, California, United States of America; 3 Department of Quality Management and Process Innovation, Academic Medical Center, University of Amsterdam, Amsterdam, The Netherlands; 4 Department of Internal Medicine, Academic Medical Center, University of Amsterdam, Amsterdam, The Netherlands; 5 Department of Pediatrics, Academic Medical Center, University of Amsterdam, Amsterdam, The Netherlands; The Royal College of Physicians and Surgeons of Canada, Canada

## Abstract

**Background:**

Tools for the evaluation, improvement and promotion of the teaching excellence of faculty remain elusive in residency settings. This study investigates (i) the reliability and validity of the data yielded by using two new instruments for evaluating the teaching qualities of medical faculty, (ii) the instruments' potential for differentiating between faculty, and (iii) the number of residents' evaluations needed per faculty to reliably use the instruments.

**Methods and Materials:**

Multicenter cross-sectional survey among 546 residents and 629 medical faculty representing 29 medical (non-surgical) specialty training programs in the Netherlands. Two instruments—one completed by residents and one by faculty—for measuring teaching qualities of faculty were developed. Statistical analyses included factor analysis, reliability and validity exploration using standard psychometric methods, calculation of the numbers of residents' evaluations needed per faculty to achieve reliable assessments and variance components and threshold analyses.

**Results:**

A total of 403 (73.8%) residents completed 3575 evaluations of 570 medical faculty while 494 (78.5%) faculty self-evaluated. In both instruments five composite-scales of faculty teaching qualities were detected with high internal consistency and reliability: learning climate (Cronbach's alpha of 0.85 for residents' instrument, 0.71 for self-evaluation instrument, professional attitude and behavior (0.84/0.75), communication of goals (0.90/0.84), evaluation of residents (0.91/0.81), and feedback (0.91/0.85). Faculty tended to evaluate themselves higher than did the residents. Up to a third of the total variance in various teaching qualities can be attributed to between-faculty differences. Some seven residents' evaluations per faculty are needed for assessments to attain a reliability level of 0.90.

**Conclusions:**

The instruments for evaluating teaching qualities of medical faculty appear to yield reliable and valid data. They are feasible for use in medical residencies, can detect between-faculty differences and supply potentially useful information for improving graduate medical education.

## Introduction

The quality of current and future health care delivery is mainly dependent on the quality of graduate medical education (GME) [1]–[4]. In many western health care delivery systems, GME is now being reformed to be more responsive to changing societal needs and health care delivery systems. Various organizations such as the Royal Society of Physicians and Surgeons of Canada (RCPSC), the American College of Physicians (ACP), the American Association of Program Directors in Internal Medicine (APDIM), the British General Medical Council (GMC) and the Dutch Central College of Medical Specialists (CCMS) involved in GME in Northern America and Europe have published their directives, position papers or recommendations for educational reform [5]–[10]. These reform proposals all stress the explicit (expanded) responsibilities of program leaders for the oversight of their teaching programs' quality, including faculty performance. In striving to maintain high quality teaching programs, faculty (self-)evaluation is no longer controversial at most teaching centers. Both feedback from residents and self-evaluation are recognized mechanisms for identifying weaknesses and strengths, and have been shown to be effective in enhancing performance [11]–[19]. However, in the face of rapid change such as the introduction of competency-based residency training, the development of effective means of faculty (self-)evaluation is a real concern. Effective evaluation entails the use of scientifically sound and practically feasible measurement instruments and processes. It also entails faculty's reflection on the evaluation results, preferably with others [19], [20], followed by tailor-made individual enhancement trajectories [21], [22].

Although validated evaluation instruments have been published over the years [23], [24], they cannot and should not be used indiscriminately in both new and old settings without relevant revalidation and updating. Recent psychometric studies underscore the importance of viewing validation as an ongoing process [25]–[27]. Measurement instruments need to be validated and updated for their continuous use in the various local, cultural and educational contexts as well as for specific groups. More importantly, any such instruments should be embedded in an effective and efficient system of feedback, support and learning.

In order to help fill the gap on reliable and valid instruments for faculty's teaching qualities embedded in an appropriate system of feedback, support and learning, we developed a new system, named System for Evaluation of Teaching Qualities, or SETQ, to support both residents' and self-evaluation of medical faculty. The (formative) core aim of the SETQ system is to increase faculties' insight in teaching performance for the purpose of self-directed learning, and ultimately, improving teaching skills in graduate medical education. In the SETQ, increased insight among faculty is achieved by annually receiving feedback from residents and by self-evaluating one's own teaching performance. Briefly, the SETQ initiative comprises four components: (i) a web-based residents' evaluation of faculty, (ii) a web-based self-evaluation by faculty, (iii) individualized faculty feedback, and (iv) individualized faculty follow-up support [28]–[30]. From a methodological perspective, combining various assessment methods should lead to more valid multi-source assessment of performance in real settings [31], [32]. The success of an integrated system such as the SETQ will depend on the separate and combined properties and impact of the system components. Hence, each component requires careful assessment of its properties. This paper focuses on the first two components of the SETQ by exploring the properties of the two evaluation instruments used in the system. More concretely, this paper aims to: (a) explore the reliability and validity of data yielded by using the two instruments underlying the SETQ for medical faculty; (b) investigate the between-faculty differentiating abilities of the SETQ instruments by quantifying the extent to which the instruments detect between-faculty differences; and (c) determine the feasibility of deploying SETQ in terms of the number of residents' evaluations per faculty needed for reliable feedback.

## Materials and Methods

### System for Evaluation of Teaching Qualities (SETQ)

We first place this study in context by describing the SETQ system. The SETQ system was initially developed in the anesthesiology department of a large academic medical center in the Netherlands [28], [33]. Based on its successful launch and positive feedback, SETQ was later offered to other clinical departments-and other hospitals-interested in assessing and improving the teaching qualities of faculty members. The introduction of SETQ to other clinical departments included the development of specialty-specific modules. Three years after its introduction, SETQ is now being used by almost 150 residency training programs in 31 teaching hospitals. Approximately 1800 faculty and 1700 residents are now involved in the continuous, longitudinal (self-)evaluation of teaching qualities of faculty. The SETQ is typically implemented within residency programs in three phases. During the first phase, data on teaching qualities are collected using two web-based instruments, one for evaluation of faculty by residents, and another for self-evaluation by faculty. In the second phase, individualized feedback reports are generated for each faculty displaying the outcomes of both types of evaluations. The averaged outcomes of colleagues are reported for reference purposes. The third phase, which is not mandatory for all training programs, involves discussing the individualized reports with each individual faculty and head of department. The aim of the discussion is to facilitate acceptance of the feedback and, if needed, define concrete steps towards improvement. Aggregated residency program level results are used to discuss each program's strengths and weaknesses.

### Study Population and Setting

From September 2008 to June 2010, 16 hospitals offered SETQ participation to 546 residents and 629 faculty of 29 medical (non-surgical) specialty training programs. All medical residents and teaching faculty were invited via electronic mail to participate in the SETQ evaluations. The invitation emphasized the formative purpose and anonymous use of the evaluations. Residents were instructed to evaluate only faculty they had been sufficiently exposed to during their training so far. Residents chose which and how many faculty to evaluate. Each faculty could only self-evaluate. The two evaluation instruments were made electronically accessible via a dedicated SETQ web portal protected by a password login. Automatic email reminders were sent after 10 days, 20 days and on the day before closing the data collection period. At clinical meetings, the training program director and/or department head encouraged faculty and residents to participate in the anonymous SETQ evaluations. The data collection lasted one month.

### The Two Instruments

The development of the SETQ instruments for medical faculty, like that of anesthesiology [16], was based on the widely used 26-item Stanford Faculty Development Program (SFDP26) questionnaire [34]–[36]. The SFDP26 is used mostly in Northern American settings, but with few recent published studies on its properties in the last ten years [35]–[37]. The SFDP26 was based on educational and psychological theories of learning and empirical observations of clinical teaching, and was found to evaluate seven categories of clinical teaching. Many of the core items in the medical SETQ instruments were based on the SFDP-26. The details of the initial instrument adaptation and development involving translations, rounds of discussions, and a specialty taskforce are described elsewhere [28], [33]. Our recent studies showed that the adapted instruments provide reliable and valid evaluations of teaching qualities of faculty in a major academic medical center [28]–[30], [33]. Through a process of consulting faculty and residents we developed two instruments per specialty: one resident-completed and one faculty self-evaluation instrument. The length of the instruments varied per specialty and could be up to 33 items. Although the instruments were specialty-specific due to the addition of supplemental items, they all shared 23 core items. Each core item had a 5-point Likert-type response: strongly disagree, disagree, neutral, agree, strongly agree. Each instrument also included two global ratings that are not part of the SETQ core items. The ratings addressed ‘faculty being seen as role model medical specialist’ and ‘faculty's overall teaching quality’ respectively. The global rating ‘faculty being seen as role model medical specialist’ had the same response scale as the 23 core items. For the global rating ‘faculty's overall teaching quality’, the 5-point Likert-type response was 1 (poor), 2 (fair), 3 (average), 4 (good), and 5 (excellent).

### Analytical Strategies

To address the aforementioned three main objectives, four main groups of analysis were conducted. First, descriptive statistics were calculated to describe the participating residents and faculty. Second, to address the first objective of this study, that is, the reliability and validity of the residents-completed and faculty-completed SETQ instruments, we conducted exploratory factor analysis, as well as reliability coefficient, item-total scale correlation, interscale correlation, and scale versus global ratings correlation analyses [27], [28]. The factor analysis used the principal components technique with Promax oblique rotation [38], [39] to explore the factor or composite-scale structure of both instruments. Although the Likert responses for the items were ordinal, we assumed the items to be interval as we expected the results to be robust to this assumption. To check for sensitivity of our findings to the interval assumption, we also re-did the factor analysis using polychoric correlation matrix that is technically more appropriate for ordinal data than the conventional Pearson's correlation matrix is. The number of extracted factors was based on the extraction criterion of eigenvalues-greater-than-1.0 from the Kaiser-Guttman rule, the result of which was subsequently triangulated by a priori specifying the number of factors to be extracted as five. Each item was assigned to the factor on which they loaded with at least a factor loading of 0.30 (to avoid low-loading items and in line with the literature). In the case of cross-factor loadings, an item was assigned to where it loaded the highest factor unless it was theoretically appropriate to leave it under the factor on which it loaded the second highest. Subsequently, each composite-scale was calculated as an average of the items that loaded the highest on it. To examine the instruments' reliability, the internal consistency reliability coefficient (Cronbach's alpha) for each scale was calculated, guided by the structuring results of the factor analysis. A Cronbach's alpha of at least 0.70 was considered satisfactory [40]. Item-total scale correlations that were corrected for item overlap (that is, eliminating the respective items one at a time from the composite-scale) were then used to check for the sensitivity of the homogeneity of the composite-scales to individual items [27]. Item-total scale correlations of 0.40 or higher were considered acceptable evidence of contribution of each item to the scale homogeneity. Inter-scale correlations for residents and faculty separately were used to check for the interpretability of the composite-scales as distinct albeit correlated constructs (for correlations ≤0.70) [27]. To explore the construct validity of the instruments further, we estimated the correlations between the composite-scales and the two global ratings, ‘faculty being seen as role model internists’ and ‘faculty's overall teaching quality’. This convenient validation approach was aimed at yielding preliminary results as part of what is envisaged as an ongoing cumulative exercise that will be updated in subsequent work and over time [27], [28], [30]. We hypothesized that faculty that received higher composite-scale scores would receive similarly higher global ratings, thus leading to higher correlations. Here, we applied both Pearson's (parametric) and Spearman's (nonparametric) correlations to check the robustness of treating the composite-scale scores as interval variables while the global ratings were ordinal. As has been reported elsewhere [27], [28], correlations of 0.40 to 0.80 between the scales and global ratings were considered appropriate.

Third, to quantify the extent to which the instruments differentiated between faculty, we used variance components decomposition from the cross-classified multilevel regression modeling of our hierarchical data [27], [41], to separate out what percentages of the total variance in each composite-scale score and each related item score were possibly due to between-faculty differences. Each percentage of the total variance possibly attributable to between-faculty differences was also recalculated after excluding the residual score-level variance. This recalculation allowed for the quantification of the percentage of the combined resident-, faculty-, program- and hospital-level variance that was due to only between-faculty differences after removing residual ‘unexplainable’ variance. Furthermore, using a threshold score of 3.5, we also estimated the percentage of faculty who were scored below 3.5 on each item and composite-scale. The threshold was set as a subjective cut-point reflecting our knowledge of the median in the frequently skewed data from our educational assessments [27]. Beyond detectable between-faculty differences, this last analysis was aimed at producing some steering information by giving insight into improvement opportunities at the faculty group level. Individual faculty enhancement goals can be set regardless of this or any absolute score.

Fourth and finally, we tackled the objective of estimating the number of residents' evaluations per faculty needed for reliable assessment and feedback using published methods [27], [28], [41]–[43]. We estimated that, in order to achieve the reliability levels comparable to those in this study, any future evaluations must have per-faculty sample sizes proportional to those observed here. Hence, for target reliability coefficients smaller (or larger) than those observed here, the number of residents' evaluations needed per-faculty should be smaller (or larger) than was actually observed. In line with previous work [29], the estimation was repeated for reliability levels of 0.60, 0.70, 0.80 and 0.90. As sensitivity analysis to cross-check our estimates based on traditional formulas, we re-estimated the reliability (Cronbach's alpha) of each composite-scale of the residents' SETQ instrument for different numbers of residents' evaluations per faculty, namely 2 to 4, 5 to 8, 9 to12 and more than 12 evaluations per faculty.

All analyses were conducted using the general-purpose statistical softwares PASW Statistics version 18.0.0 for Mac (IBM SPSS Inc, 2009), SAS version 9.2 (SAS Institute, Cary, NC, 2008), and Microsoft Excel 2008 for Mac version 12.2.6 (Microsoft Corporation, 2007). Although under Dutch law institutional review board approval was not required for this study we have taken all necessary precautions to guarantee and protect the anonymity and confidentiality of our study participants, including written consent to the use of the data for research purposes by the SETQ research group at the Academic Medical Center of the University of Amsterdam (AMC). Researchers do not have access to data identifying individual SETQ participants.

## Results

### Study Participants

Table 1 shows the characteristics of the participating residents and faculty. In total, 403 residents from every residency year and 494 faculty members participated in the study yielding response rates of 73.8% and 78.5% respectively. Residents evaluated 570 (91%) of all faculty, yielding a total of 3,575 evaluations or about 6.2 evaluations per faculty on average.

**Table 1:** Characteristics of residents and medical faculty who participated in the SETQ evaluations.

Specialties#	All medical specialties	IM*	C	N	P	R	RT	CG	PA	NM	PRM	PSY
Number of hospitals	16	3	1	3	11	1	2	1	1	1	1	2
Number of training programs	29	5	1	3	11	1	2	1	1	1	1	2
Number of residents invited/number that participated (% participation)	403/546 (73.8)	100	16	61	129	18	15	5	9	6	4	40
Total number of residents' evaluations	3575	912	177	560	1029	341	135	42	97	30	22	230
Number of faculty invited/participated (% participation)	629/494 (78.5)	98	22	48	227	24	26	7	9	5	7	21
Total number of faculty actually evaluated by residents (including faculty who did not self-evaluate)	570	124	23	52	253	25	32	10	14	5	7	25
Mean number of faculty evaluated by each resident	8.9	9.1	11.1	9.2	8.0	18.9	9.0	8.4	10.8	5.0	5.5	5.8
Mean number of residents evaluations per faculty	6.3	7.4	7.7	10.8	4.1	13.6	4.2	4.2	6.9	6.0	3.1	9.2
Mean number years of practice since first registration as medical specialist	12.1	13.6	11.1	13.1	11.7	11.2	11.5	10.6	9.3	10.6	12	12.6
Percentage of faculty who had formal training as clinical educators	50.2	69	18.2	39.6	61.7	50	19.2	85.7	33.3	100	57.1	47.6

# IM = Internal medicine; C = cardiology; N = neurology; P = pediatrics; R = radiology; RT = radiotherapy; CG = clinical genetics; PA = pathology; NM = nuclear medicine; PRM = physical rehabilitation Medicine; PSY = psychiatry.

*Includes chest medicine and gastroenterology.

### Reliability and Validity of the Resident and Faculty SETQ Instruments

Table 2 gives an overview of the factor loadings, Cronbach's alpha, and corrected item-total correlations for both instruments separately. The factor analysis yielded five composite-scales of faculty's teaching qualities: ‘learning climate’ (items L1 to L7), ‘professional attitude and behavior towards residents’ (items P1 to P3), ‘communication of goals’ (items C1 to C5), ‘evaluation of residents’ (items E1 to E4), and ‘feedback’ (items F1 to F4). The factor loadings in the resident analysis were all above 0.70, except for three items in the scale ‘learning climate’ which still loaded as high as 0.60 (L1) and 0.59 (L2, L3). In the faculty instrument, four of the constructs achieved good overall factor loadings (0.67–0.88). ‘Learning climate' contained three items (L3, L4, L7) with lower factor loadings (0.24, 0.33, and 0.44 respectively) in the faculty instrument. For both instruments, the additional factor analysis based on the polychoric correlation matrix yielded factor loadings higher than, yet similar to, those based on the conventional Pearson's correlation matrix. Both approaches yielded the same factor structure, hence essentially the same conclusion.

**Table 2:** Item and scale characteristics, internal consistency reliability, and item-total correlations.

Item nr	Scale and items†	Factor loadings on primary scale‡		Internal consistency reliability: Cronbach's α		Corrected item-total correlations	
		Residents' evaluations	Faculty self-evaluation	Residents' evaluations	Faculty self-evaluation	Residents' evaluations	Faculty self-evaluation
	*Learning climate*		0.85	0.71		
L1	Encourages residents to participate actively in discussions	0. 60 (0.58)	0.62 (0.81)			0.67	0.46
L2	Stimulates residents to bring up problems	0.59 (0.48)	0.61 (0.72)			0.68	0.50
L3	Teaches residents time management	0.59 (0.57)	0.24^a^ (0.30)			0.54	0.33
L4	Keeps to teaching goals; avoids digressions	0.71 (0.78)	0.33 (0.32)			0.55	0.27
L5	Motivates residents to study further	0.79 (0.73)	0.79 (0.79)			0.71	0.58
L6	Stimulates residents to keep up with the literature	0.78 (0.72)	0.81 (0.77)			0.62	0.47
L7	Prepares well for teaching presentations and talks	0.73 (0.78)	0.44 (0.40)			0.56	0.36
	*Professional attitude towards and support of residents*			0.84	0.75		
P1	Listens attentively to residents	0.87 (0.90)	0.78 (0.86)			0.74	0.58
P2	Is respectful towards residents	0.87 (0.90)	0.81 (0.89)			0.75	0.65
P3	Is easily approachable during on-calls	0.77 (0.81)	0.69 (0.82)			0.64	0.53
	*Communication of goals*			0.90	0.84		
C1	States learning goals clearly	0.90 (0.93)	0.84 (0.90)			0.83	0.73
C2	States relevant goals	0.92 (0.94)	0.88 (0.91)			0.86	0.77
C3	Prioritizes learning goals	0.92 (0.95)	0.86 (0.91)			0.86	0.74
C4	Repeats stated learning goals periodically	0.91 (0.94)	0.83 (0.90)			0.85	0.72
C5	Offers to conduct mini-CEX (clinical examination exercise) regularly	0.60 (0.64)	0.53 (0.44)			0.46	0.38
	*Evaluation of residents*			0.91 (0.92)	0.81		
E1	Evaluates residents' specialty knowledge regularly	0.89 (0.91)	0.79 (0.86)			0.83	0.63
E2	Evaluates residents' analytical abilities regularly	0.88 (0.91)	0.84 (0.90)			0.81	0.67
E3	Evaluates residents' application of knowledge to specific patients regularly	0.90 (0.92)	0.83 (0.86)			0.86	0.71
E4	Evaluates residents' medical skills regularly	0.79 (0.81)	0.67 (0.74)			0.71	0.53
	*Feedback*			0.91	0.85		
F1	Regularly gives positive feedback to residents	0.74 (0.77)	0.47^b^ (0.69)			0.68	0.54
F2	Gives corrective feedback to residents	0.91 (0.92)	0.81 (0.87)			0.80	0.71
F3	Explains why residents are incorrect	0.93 (0.94)	0.85 (0.92)			0.87	0.80
F4	Offers suggestions for improvement	0.91 (0.93)	0.80 (0.93)			0.85	0.74

† The items shared the same subject ‘During my residency in [medical specialty], my attending generally…’ (residents' evaluation of faculty) or ‘In my role as an attending internist/faculty, I generally…’ (faculty self-evaluation).

‡ Factor loadings in parentheses were obtained using the polychoric correlation matrix as input for the principal components analysis. Similar results but with even higher factor loadings were also obtained when we applied maximum likelihood as the factor estimation technique.

§ Total variance explained by all 5 domains of teaching qualities: 73.08% among residents and 66.39% among faculty

a The item L3 also loads (0.56) on the scale ‘Communication of goals’ in the self-evaluation.

b The item F1 also loads (0.70) on the scale ‘Professional attitude and behavior towards residents’ in the self-evaluation.

Cronbach's alpha for all 23 items combined: 0.95 on the resident evaluation (0.96 when aggregated across faculty) and 0.91 on the self-evaluation.

In the residents' instrument, Cronbach's alpha was above 0.84 for each composite-scale. For the faculty instrument, Cronbach's alpha was 0.74 or higher for the five scales. In both instruments, the item-total correlations were all above 0.40 for all items within their respective scales, with the exception of three items (L3, L4, L7) that had item-total correlations of 0.33, 0.27, and 0.36 respectively with ‘learning climate’ in the faculty instrument.

For the residents' instrument, the inter-scale correlations ranged from 0.37 (*P*<0.001) between ‘professional attitude and behavior towards residents’ and ‘evaluation of residents’ to 0.61 (*P*<0.001) between ‘learning climate’ and both ‘evaluation of residents’ and ‘communication of goals’ (Table 3). For the faculty instrument, the inter-scale correlations ranged from 0.25 (*P*<0.001) between ‘professional attitude towards residents’ and ‘communication of goals’ to 0.56 (*P*<0.001) between ‘learning climate’ and ‘feedback’.

**Table 3:** Inter-scale correlations for residents' and faculty evaluations separately.

	Learning climate	Professional attitude and behavior towards residents	Communication of goals	Evaluation of residents	Feedback
*Residents*' *evaluation of faculty*					
Learning climate	1	0.49	0.61	0.61	0.59
Professional attitude and behavior towards residents		1	0.38	0.37	0.49
Communication of goals			1	0.58	0.55
Evaluation of residents				1	0.55
Feedback					1
*Faculty self-evaluation*					
Learning climate	1	0.41	0.51	0.54	0.56
Professional attitude and behavior towards residents		1	0.25	0.30	0.42
Communication of goals			1	0.47	0.45
Evaluation of residents				1	0.50
Feedback					1

All correlation coefficients have two-tailed *P*<0.01 unless stated otherwise.

Table 4 displays the results of validation of the scales by way of their theoretically expected correlations with two global ratings ‘faculty being seen as role model medical specialist’ and ‘overall teaching quality’. For the residents’ instrument, the composite-scales exhibited correlations ranging from 0.48 to 0.61 with global rating ‘faculty being seen as role model medical specialist’ and ‘overall teaching quality’. The correlations were somewhat higher for the global rating ‘overall teaching quality’. For the faculty instrument, the correlations with both global ratings were in the ranges 0.35 to 0.48 and 0.29 to 0.48 respectively.

**Table 4:** Parametric (nonparametric) correlations between scales and global ratings of (i) faculty being seen as a role model medical specialist and (ii) faculty's overall teaching quality, estimated separately for residents’ and faculty's evaluations.

Scales	Faculty seen as role model medical specialist		Faculty's overall teaching quality	
	Residents' evaluations	Faculty self-evaluation	Residents' evaluations	Faculty self-evaluation
Learning climate	0.61 (0.62)	0.48 (0.46)	0.68 (0.70)	0.48 (0.55)
Professional attitude towards residents	0.61 (0.60)	0.35 (0.42)	0.61 (0.58)	0.29 (0.39)
Communication of goals	0.48 (0.50)	0.36 (0.30)	0.57 (0.58)	0.43 (0.38)
Evaluation of residents	0.51 (0.51)	0.41 (0.42)	0.57 (0.58)	0.42 (0.42)
Feedback	0.61 (0.61)	0.40 (0.36)	0.67 (0.67)	0.39 (0.40)

Pearson's (Spearman's) correlations are reported respectively outside and inside the parenthesis. All correlation coefficients have two-tailed *P*<0.001 unless stated otherwise.

### Differentiating Between Individual Faculty Performance

Table 5 shows the results on how well the instruments differentiated between faculty. For contextualization purposes, the first part of table 5 shows the median scores for the five teaching scales and their 23 items as well as the 20th and 80th percentile scores. On a scale of 1 to 5, faculty evaluated themselves highly, with their median scale scores ranging from 3.00 for ‘communication of goals’ to 4.00 for ‘professional attitude towards residents’ and 4.00 for ‘feedback’. Residents evaluated their faculty with scores ranging from 3.12 for ‘communication of goals’ to 4.07 for ‘professional attitude towards residents.’

**Table 5:** Scale mean scores, item median scores, and measure of between-faculty differences based on residents' and self-evaluation of faculty.

Item nr	Scale and items	Median score (20^th^–80^th^ percentile range)		Percentage of total variance due to between-faculty differences in the residents' evaluations	Percentage of combined resident-faculty-specialty-hospital variance* that is due to between-faculty differences in the residents' evaluations	Percentage of faculty scoring below 3.5 on a scale of 1 to 5 on the residents' evaluation
		Faculty self-evaluation	Residents' evaluations			
	*Learning climate*	*3.71 (3.29*–*4.00)*	*3.59 (3.14*–*4.00)*	*24.2*	*47.3*	*38*
L1	Encourages residents to participate actively in discussions	4.00 (3.00–4.00)	4.00 (3.50–4.38)	20.5	53.4	16
L2	Stimulates residents to bring up problems	4.00 (3.00–4.00)	4.00 (3.50–4.33)	21.6	57.0	18
L3	Teaches residents time management	4.00 (3.00–4.00)	3.25 (2.85–4.79)	17.7	39.6	58
L4	Keeps to teaching goals; avoids digressions	4.00 (3.00–4.00)	4.00 (3.35–4.22)	26.1	64.6	24
L5	Motivates residents to study further	4.00 (3.00–4.00)	4.00 (3.50–4.35)	21.6	55.2	16
L6	Stimulates residents to keep up with the literature	3.00 (3.00–4.00)	3.74 (3.20–4.50)	20.4	44.6	32
L7	Prepares well for teaching presentations and talks	4.00 (4.00–5.00)	4.14 (3.67–4.50)	22.9	54.3	12
	*Professional attitude towards and support of residents*	*4.00 (4.00*–*4.67)*	*4.07 (3.34*–*4.54)*	*30.3*	*53.9*	*23*
P1	Listens attentively to residents	4.00 (4.00–5.00)	4.20 (3.60–4.61)	28.9	65.9	14
P2	Is respectful towards residents	4.00 (4.00–5.00)	4.40 (3.88–4.75)	27.5	63.4	7
P3	Is easily approachable during on-calls	4.00 (4.00–5.00)	4.50 (4.00–4.86)	29.9	66.0	8
	*Communication of goals*	*3.00 (2.40*–*3.60)*	*3.12 (2.66*–*3.53)*	*16.7*	*32.8*	*73*
C1	States learning goals clearly	3.00 (3.00–4.00)	3.50 (3.00–4.00)	17.0	34.5	44.9
C2	States relevant goals	3.00 (2.80–4.00)	3.43 (3.00–4.00)	16.6	32.5	50.7
C3	Prioritizes learning goals	3.00 (2.00–4.00)	3.33 (3.00–3.84)	13.2	26.5	58.6
C4	Repeats stated learning goals periodically	3.00 (2.00–4.00)	3.33 (2.92–3.80)	14.0	28.8	60.2
C5	Offers to conduct mini-CEX (clinical examination exercise) regularly	3.00 (2.00–4.00)	2.5 (2.00–3.13)	13.9	24.0	85.7
	*Evaluation of residents*	*3.50 (3.00*–*4.00)*	*3.60 (3.13*–*4.00)*	*20.9*	*45.0*	*38*
E1	Evaluates residents' specialty knowledge regularly	4.00 (3.00–4.00)	3.75 (3.33–4.19)	22.6	47.8	29
E2	Evaluates residents' analytical abilities regularly	4.00 (3.00–4.00)	3.80 (3.33–4.20)	20.7	48.3	26
E3	Evaluates residents' application of knowledge to specific patients regularly	4.00 (3.00–4.00)	3.84 (3.38–4.20)	20.4	46.1	23
E4	Evaluates residents' medical skills regularly	3.00 (3.00–4.00)	3.50 (3.00–4.00)	15.8	44.1	46
	*Feedback*	*4.00 (3.50*–*4.00)*	*3.80 (3.32*–*4.17)*	*16.2*	*40.7*	*25*
F1	Regularly gives positive feedback to residents	4.00 (3.00–4.00)	4.00 (3.35–4.31)	23.5	59.5	25
F2	Gives corrective feedback to residents	4.00 (3.00–4.00)	3.92 (3.44–4.25)	16.2	39.2	20
F3	Explains why residents are incorrect	4.00 (3.00–4.00)	3.89 (3.40–4.25)	16.9	44.1	21
F4	Offers suggestions for improvement	4.00 (3.00–4.00)	3.88 (3.40–4.29)	17.0	44.1	21

*This is equivalent to the total variance in the item or scale but without the residual (score-level) variance component. This quantifies the contribution of between-faculty differences in residents' evaluation combined.

Further, table 5 reports the results of the variance components analysis to determine how much of the variation was due to between-faculty differences per scale and item. The third column shows that about 16% (‘feedback to residents’) to 30% (‘professional attitude’) of the total variance in the composite-scales can be attributed to between-faculty differences. Upon exclusion of the residual variance, these percentages increased to 41% for ‘feedback’ and 54% for ‘professional attitude’ (column 4 of Table 5). These numbers are higher for some individual items that load on each composite-scale. Finally, the last column displays the percentage of faculty evaluated below the pre-defined performance level of 3.5. The item where most (85.7%) faculty did not reach the threshold was item C5, ‘Offers to conduct mini-CEX (clinical examination exercise) regularly’. Only 7% was evaluated as not reaching 3.5 on item P2 (‘is respectful to residents’). There were wide variations across scales and items in the percentage of faculty evaluated by residents as scoring below 3.5.

### Number of Residents' Evaluations Per Faculty Needed

For producing reliable feedback reports at various levels of reliability, we found that, for each of the 5 teaching qualities, 4 residents' evaluations are needed to achieve reliability of at least 0.60. To achieve a reliability level of 0.70 or 0.80 a minimum number of 5 respectively 6 residents' evaluations is required. For a reliability of 0.90, 7 residents' evaluations per faculty appear adequate. (Tables 6 and 7).

**Table 6:** Number of residents' evaluations needed per faculty for reliable evaluation of faculty's teaching qualities.

Scales	Cronbach's alpha of 0.60	Cronbach's alpha of 0.70	Cronbach's alpha of 0.80	Cronbach's alpha of 0.90
Learning climate	4	5	6	7
Professional attitude towards residents	4	5	6	7
Communication of goals	4	5	6	7
Evaluation of residents	4	5	6	7
Feedback	4	5	6	7

**Table 7:** Estimated reliabilities (Cronbach's alphas) at different numbers of residents' evaluations completed per faculty (based on residents' evaluations aggregated to the level of the faculty).

Scales	2–4 evaluations per faculty	5–8 evaluations per faculty	9–12 evaluations per faculty	>12 evaluations per faculty
Learning climate	0.88	0.86	0.89	0.90
Professional attitude towards residents	0.90	0.89	0.89	0.90
Communication of goals	0.90	0.90	0.90	0.92
Evaluation of residents	0.91	0.93	0.93	0.94
Feedback	0.94	0.92	0.92	0.94
Overall instrument	0.96	0.96	0.96	0.98

## Discussion

### Main Findings

This study found that the two instruments underlying the SETQ system seemed reliable and valid for the evaluation of the teaching qualities of medical faculty within residency training programs. Residents' evaluations could differentiate between high and low performing teaching faculty. High proportion of the total variance could be attributed to between-faculty differences, indicating possible roles for faculty-specific factors as explanations. Finally, for reliable [27], [28], [41] assessment of medical faculty, we found that 4 to 7 residents' evaluations per faculty were needed to achieve reliability coefficients of 0.60 to 0.90. This would be attainable for most medical residency training programs as we observed in our study.

### Limitations and Sensitivity Analysis

Before discussing the findings, a few limitations of this study should be explored. First, the cross-sectional design of this study does not support assessment of test-retest reliability. However, the high levels of inter-rater reliability found here suggest that the intra-observer reliability can only be higher [27], [28]. Second, the findings presented here may not be generalizable to surgical residents and faculty since those residency programs may have their own structures and cultures. Work is currently being done to replicate the findings of our studies in surgical settings. Finally, in some places such as in the factor analysis and the correlation of composite-scales with global ratings we treated ordinal variables as interval because we expected our parametric analysis of ordinal data to remain robust [44]–[52]. Indeed, this was the case as can be seen in Tables 2 and 4. In particular, although it is appropriate to use a polychoric correlation matrix for the factor analysis of ordinal data, our finding that the factor analysis based on the more appropriate polychoric correlation matrix yielded higher but similar factor loadings and factor structure as that based on the commonly used Pearson's correlation matrix is reassuring but not surprising. This finding that the two results reached essentially the same conclusion is in line with the well-documented remarkable robustness of the Pearson's correlation and of other related parametric methods when applied to settings where their assumptions were violated [44]–[52].

### Explanation of Results

Residency programs are increasingly defined in terms of what is expected from residents by the end of their training [3], [53]. This shift towards competency-based residencies requires clinical teachers to review, reorient and potentially improve their teaching qualities. Our study showed that the SETQ instruments developed can be adapted for the systematic evaluation of medical faculty responsible for training their future colleagues. This study provides empirical support for the reliability and validity of the results obtained from the residents- and self-completed instruments for medical faculty evaluations. Compared to the SETQ instruments developed for anesthesiology faculty [28], [33] the 23-item medical SETQ instruments show slightly better qualities. The results of the reliability and validity analysis indicate that we could tap into five domains seen as relevant aspects of teaching by both residents and faculty. We observed that two items (L3 and L4) show low factor loadings and corrected item-total correlations in the faculty-completed instrument. In two other smaller studies [28], [33] we reported similar findings suggesting that it may reflect faculty's different perception of teaching compared to residents. In the original SFDP26 instrument, these two items were on a separate scale (named ‘control of session’); however, this instrument was not administered to faculty but to residents only. Given the ambiguous findings, we decided to maintain the items in both instruments but will continue to study the uniqueness of the problematic items L3 and L4 in future research.

Based on the finding that good clinician-educators make good role model medical specialists for residents [54]–[56], the correlations of each of the five composites or domains with the global rating of being seen as role model medical specialists offer intuitive support for the five teaching domains as part of the phenomenon of clinical teaching (Table 4). If the five composites addressed various teaching qualities and the one-item global rating on overall teaching quality did so similarly, then we could expect the composites to correlate at least moderately with the global rating (as indeed was the case). The latter correlations should not, however, be too high (for example, greater than 0.80) because that would point to redundancy of the entire instrument [27], [28]. That is, excessively high correlations of more than 0.80 could imply that the entire 23-item instrument could be reduced to only one or two global items. Our findings of correlations less than 0.80 (actual results ranging from 0.25 to 0.61) provide some additional, hypothesis-based (construct) validation of the SETQ instruments.

Part of the educational reforms going on worldwide is the transition from faculty being ‘merely’ clinical experts to faculty becoming all-round professionals [5], [57], including being high performing teachers, supervisors and role models for their future colleagues. Our study showed that not all teaching faculty were performing at the same high level yet. Residents' evaluations exposed the differences between individual clinician-educators. For the various teaching scales and items, residents-of-faculty scores varied by up to two points on the relatively narrow five point instrument. Clearly, there is room for improvement for individual faculty–in fact for all individual faculty scoring less than the perfect 5-particularly since the reported variance can be ascribed for a great part to differences caused by factors related to faculty's behavior, attitude or characteristics. As part of the SETQ system faculty should reflect on their individual feedback results, preferably facilitated by program leaders since guided reflection is more effective in achieving change [21], [22]. Next, improvement goals when appropriate should be defined and pursued. Many teaching hospitals have mechanisms in place to assist faculty to achieve advancement, including faculty development programs [22], [58]–[61]. Understandably, this requires supportive institutional leadership, appropriate resource allocation, and recognition for teaching excellence [57]. In addition, program leaders may want to map the program's strengths and weaknesses for priority setting and policymaking by defining (minimum or optimal) performance level expectations for each faculty. We illustrated how this would turn out when the SETQ performance level was targeted at 3.50 (Table 5). In the Netherlands, where a formative approach is favoured [62], clinician-educators who do not pass the preset teaching standard would then be encouraged and supported to improve their performance. In more summative contexts, where trainee evaluations are often considered the most important performance measure [63] the SETQ results could be part of faculty's promotion or reward systems.

### Implications for Clinical Education, Research and Policy

Good clinical teachers are indispensable to academic medical centers as they contribute to excellence in patient care and medical training. The increased public demand for excellence and the introduction of competency-based residencies should drive the development of formative systems that facilitate the continuous improvement of teaching performance. One such formative system is the SETQ. The demonstrated results of the SETQ instruments could also support use in a more summative context. The SETQ was built to support faculty in their self-directed learning efforts, assuming that motivation for professional development remains a priority (acquired or inherent) characteristic of physicians. Anecdotal reports from faculty confirm that they do discuss their feedback with program leaders, peers, and/or family members and that the individualized feedback reports have increased their awareness about their teaching qualities. Residents claimed they observed improved teaching performance and our preliminary studies seem to confirm these claims (unpublished internal report). Our current studies aim at determining whether resident-of-faculty feedback and faculty self-evaluations improve clinical teaching.

Clearly, SETQ is and should be a dynamic system. Future research will have to focus on explaining and reducing the variation in teaching qualities between faculty members with the objective of also improving teaching abilities of clinician-educators. Ultimately, research should be conducted to investigate the impact of teaching qualities on residents' and patients' outcomes.

### Conclusions

The SETQ instruments seem to yield reliable and valid measurements and could reasonably be implemented in medical residencies. The instruments have good between-faculty differentiating abilities. Faculty feedback seems useful for increasing awareness and designing faculty development tracks, both at individual and group levels. This study went further than previous work by including the voice of the faculty in self-evaluating teaching qualities in order to support self-directed learning.
